# Drug-Target Interaction Prediction via Dual Laplacian Graph Regularized Matrix Completion

**DOI:** 10.1155/2018/1425608

**Published:** 2018-12-02

**Authors:** Minhui Wang, Chang Tang, Jiajia Chen

**Affiliations:** ^1^Department of Pharmacy, People's Hospital of Lian'shui County, Huai'an 223300, China; ^2^School of Computer Science, China University of Geosciences, Wuhan 430074, China; ^3^Department of Pharmacy, The Affiliated Huai'an Hospital of Xuzhou Medical University, Huai'an 223002, China

## Abstract

Drug-target interactions play an important role for biomedical drug discovery and development. However, it is expensive and time-consuming to accomplish this task by experimental determination. Therefore, developing computational techniques for drug-target interaction prediction is urgent and has practical significance. In this work, we propose an effective computational model of dual Laplacian graph regularized matrix completion, referred to as DLGRMC briefly, to infer the unknown drug-target interactions. Specifically, DLGRMC transforms the task of drug-target interaction prediction into a matrix completion problem, in which the potential interactions between drugs and targets can be obtained based on the prediction scores after the matrix completion procedure. In DLGRMC, the drug pairwise chemical structure similarities and the target pairwise genomic sequence similarities are fully exploited to serve the matrix completion by using a dual Laplacian graph regularization term; i.e., drugs with similar chemical structure are more likely to have interactions with similar targets and targets with similar genomic sequence similarity are more likely to have interactions with similar drugs. In addition, during the matrix completion process, an indicator matrix with binary values which indicates the indices of the observed drug-target interactions is deployed to preserve the experimental confirmed interactions. Furthermore, we develop an alternative iterative strategy to solve the constrained matrix completion problem based on Augmented Lagrange Multiplier algorithm. We evaluate DLGRMC on five benchmark datasets and the results show that DLGRMC outperforms several state-of-the-art approaches in terms of 10-fold cross validation based AUPR values and PR curves. In addition, case studies also demonstrate that DLGRMC can successfully predict most of the experimental validated drug-target interactions.

## 1. Introduction

Identifying potential drug-target interactions (DTIs) is a challenging and meaningful step in precision medicine and biomedical research [1–8]; it is also crucial during drug discovery process. With predicted positive DTIs, one can find novel targets for existing drugs or identify targets for new drugs [9–12]. Although there are almost 30,000 human genes, only fewer than 400 of them could be used as drug targets in the treatment of diseases [13]. Therefore, identifying more DTIs is an extremely valuable task which can bring huge breakthrough in biopharmaceutical and biomedical research.

The mainly traditional and reliable methods for DTIs prediction are biochemical experiments, but these methods are very expensive and time-consuming. Thus, only a small amount of DTIs have been validated by experiments based methods. This motivates the development of computational methods for DTIs prediction. In addition, various experimental data of drugs and genes such as KEGG [14], DrugBank [15], and Genbank [16] also serve to develop computational techniques to infer the potential DTIs.

A wide variety of computational techniques for DTIs prediction have been proposed, and these techniques often rely on some machine learning models such as support vector machine (SVM) [17–20], logistic regression [21, 22] and naive Bayesian classifiers [23], matrix factorization, and kernel learning, and network inference. Bai et al. [18] applied genetic algorithm to screen related compounds, the drug-target pairs with strong binding capacity were found with SVM and particle swarm optimization. Garcia-Sosa et al. [21, 23] used logistic regression and naive Bayesian classifiers for classification of compounds. In [24], the experimental validated targets are employed to train a SVM model and find potential proteins with similar structure. Matrix factorization based methods decompose the matrix which represents the drug-target network into multiple low-rank matrices. The decomposed matrices consisting of latent features are used to exploit the drug-target interactions. The Bayesian matrix factorization [25] and collaborative matrix factorization [26] are two typical methods. In [27], Ezzat et al. added a dual Laplacian graph regularization term to the matrix factorization model for learning a manifold on which the data are assumed to lie. The typical kernel leaning methods include the pair kernel method [28], net Laplacian regularized least squares [29], and the regularized least squares with Kronecker product kernel [30]. As to network inference methods, they usually formulate the drug-target interactions prediction as a graph leaning problem. Bleakley and Yamanishi [31] proposed a novel supervised inference method to predict unknown drug-target interactions by constructing a bipartite graph; the bipartite local model first predicts target proteins of a given drug and then predicts drugs targeting a given protein. As a improved version of the bipartite local model, Mei et al. [32] considered new drug candidates through its neighbors' interaction profiles. By considering the drug-drug similarities and target-target similarities, Chen et al. [33] developed a network-based random walk with restart on the heterogeneous network to predict potential drug-target interactions. Emig et al. [13] introduced a network-based approach which integrates disease gene expression signatures and a molecular interaction network. In order to enhance the similarity measures to include nonstructural information, Shi et al. [34] introduced a new concept named “super-target" to handle the problem of possibly missing interactions. Different to existing methods which are based on the single view data, Zhang et al. [11] integrated the drug and target data from different views and proposed a multiview DTIs prediction method based on clustering. Li and Cai [35] also extended the single view low-rank representation model to multiview low-rank embedding for DTIs prediction. In [36], Zhang et al. proposed a label propagation method with linear neighborhood information for predicting unobserved drug-target interactions; the drug-drug linear neighborhood similarities are used to rank the interaction scores. A brief review of DTIs prediction can be found in [9].

**Figure 1:**
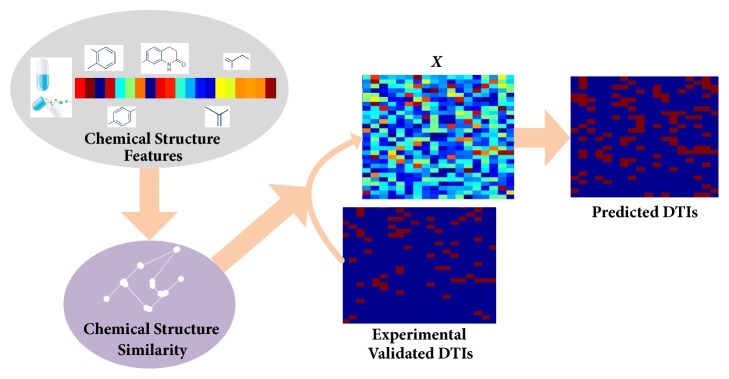
Overview of the proposed DTIs prediction method. The chemical structure similarity between drugs and the genomic sequence similarity between targets are used to serve the matrix completion. Meanwhile, the experimental validated DTIs are preserved by a binary indicator matrix.

Although there are so many methods have been proposed for DTIs prediction, the results are far from satisfactory. The key issue of this problem is how to efficiently use the existing validated DTIs and exploit the useful information hidden among drugs or targets [37]. For most of existing methods, the drug-drug similarities and target-target similarities play important roles [26–28, 31, 34, 38, 39]. Therefore, different ways for calculating drug-drug similarities have been proposed, such as cosine similarity, Gauss similarity, and Jaccard similarity. In this paper, we propose a Laplacian graph regularized matrix completion model for DTIs prediction, in which the drug-drug similarities are used to construct a similarity graph for regularizing that drugs with similar chemical structure are more likely to have interactions with similar targets and targets with similar genomic sequence similarity are more likely to have interactions with similar drugs. During the matrix completion process, the experimental validated interactions are preserved well by using an indicator matrix with binary values which indicates whether there exists validated interaction between a drug and a target. An alternative iterative strategy based on Augmented Lagrange Multiplier algorithm is developed to solve the constrained matrix completion problem. Extensive experiments on four benchmark datasets are conducted to validate the efficacy of the proposed Laplacian graph regularized matrix completion model (DLGRMC) for DTIs prediction. The architecture of our proposed method is shown in Figure 1.

## 2. Materials and Methods

### 2.1. Materials

In order to evaluate the DTIs prediction performance of the proposed DLGRMC, four small-scale benchmark datasets which correspond to four different target protein types and a large-scale dataset are used in our experiments, including nuclear receptors (NRs), G protein-coupled receptors (GPCRs), ion channels (ICs), enzymes (Es) [40], and DrugBank (DB) [41]. The former four datasets are publicly available at http://web.kuicr.kyoto-u.ac.jp/supp/yoshi/drugtarget/. The last DrugBank dataset is a unique bioinformatics and cheminformatics resource that combines detailed drug data with comprehensive drug-target information. The data used in this study was released on July 03, 2018 (version 5.1.1). The drugs and targets data were extracted from the DrugBank database website at http://www.drugbank.ca/. We only use the approved drug-target interactions in our experiments. Therefore, there are totally 1936 drugs and 1609 targets, respectively. The number of approved drug-target interactions is 7019. The approved drug structures and approved target sequences were downloaded from https://www.drugbank.ca/releases/latest#structures and https://www.drugbank.ca/releases/latest#target-sequences, respectively.

Table 1 summarizes the simple statistics of the four datasets. In Table 1, we present three types of information for each dataset, i.e., the experimental validated DTIs, the similarities between drugs, and the similarities between targets. Specifically, the validated DTIs are obtained from public datasets including BRENDA [42], KEGG BRITE [43], DrugBank [44], and SuperTarget [45]. The drug similarities are calculated via the chemical structures of the compounds, which are derived from the DRUG and COMPOUND sections in the KEGG LIGAND dataset [43]. The chemical structure similarities between compounds are computed by using SIMCOMP score [46], where SIMCOMP provides a global similarity score based on the size of the common substructures between two compounds using a graph alignment algorithm. The similarity between two compounds *c* and *c*′ is computed as *S*(*c*, *c*′) = |*c*∩*c*′ | /|*c* ∪ *c*′|. By applying this operation to all compound pairs, we can construct a drug similarity matrix. The target similarities are computed via the amino acid sequences of target proteins, which are obtained from the KEGG GENES dataset [43]. The sequence similarities between the proteins are computed by using a normalized version of Smith–Waterman scores [47]. The normalized SmithWaterman score between two proteins *g* and *g*′ is computed as $S(g,g^{\prime })=SW(g,g^{\prime })/\sqrt{SW(g,g)SW(g^{\prime },g^{\prime })}$, where *SW*(·, ·) means the original SmithWaterman score. By applying this operation to all protein pairs, we can construct a target similarity matrix.

### 2.2. Problem Formulation of DTIs Prediction

In this work, we use two sets *𝒟* = {*D*_*i*_}_*i*=1_^*d*^ and *𝒯* = {*T*_*i*_}_*i*=1_^*t*^ to denote *d* drugs and *t* targets, respectively. The experimentally validated DTIs are represented by a binary matrix *M* ∈ {0,1}^*d*×*t*^. If a drug *D*_*i*_ has been experimentally validated to interact with a target *T*_*j*_, then *M*_*ij*_ = 1; otherwise, *M*_*ij*_ = 0. The nonzero elements in *M* are called “known interaction” and can be regarded as positive observations, while the zero elements in *M* are called “unknown interaction” and can be regarded as negative observations. In addition, the drug similarities are denoted as *DS* ∈ *ℝ*^*d*×*d*^, and the target similarities are represented as *TS* ∈ *ℝ*^*t*×*t*^. The aim of DTIs prediction is to uncover the possible interactions from the negative observations by using certain prior information of drugs and targets. The candidate drug-target interactions will be chosen as predicted interactions according to their predicted probabilities in descending order.

### 2.3. Matrix Completion

Matrix completion aims to fill in the missing entries of a partially observed matrix *M*. One of the mostly used model of the matrix completion problem is to find the lowest rank matrix *X* which matches the matrix *M*, which we wish to recover, for all entries in the set *E* of observed entries. The basic mathematical formulation of this problem is as follows:

(1) minX rankXs.t. Xij=Mij∀i,j∈E.

Due to the fact that problem (1) is nonconvex and no efficient solution can be obtained, (1) is usually transformed to the following convex problem by relaxing the rank function into the nuclear norm:

(2) minX X∗s.t. Xij=Mij∀i,j∈E.

where ‖·‖_*∗*_ is the nuclear norm, which is equal to the sum of singular values of *X*. Equation (2) can be solved by using the singular value thresholding (SVT) algorithm [48].

### 2.4. Dual Laplacian Graph Regularized Matrix Completion (DLGRMC)

Supposing there are *d* drugs and *t* targets, if we use the matrix *M* ∈ *ℝ*^*d*×*t*^ to denote the drug-target interactions and denote *E* as the validated interaction set, then (2) can be directly used for potential DTIs prediction. However, the drug-drug similarities and target-target similarities which have been demonstrated useful in previous works are not fully exploited to serve the matrix completion model. Thus, we believe that the two kinds of similarities can advantage the matrix completion model; of course, better DTIs prediction results can be expected. In this work, we present a new objective function through incorporation of the drug-drug similarities and target-target similarities into the standard matrix completion framework for DTIs prediction. We use a dual Laplacian graph regularization term to constrain that drugs with similar chemical structure are more likely to have connections with similar targets and targets with similar genomic sequence similarity are more likely to have interactions with similar drugs. The optimization problem of DLGRMC can be formulated as follows:

(3) minX X∗+αXF2+βA∘X−AF2+λ∑i,j=1dxi−xj2DSi,j+∑p,q=1txp−xq2TSi,j

where *x*^*i*^ and *x*^*j*^ represent the *i*-th row and *j*-th row of *X*, respectively. *x*_*p*_ and *x*_*q*_ represent the *p*-th column and *q*-th column of *X*, respectively. *α*, *β*, and *λ* are three regularization parameters, and “∘" denotes the Hadamard product of two matrices. The Tikhonov regularization on *X* is used to ensure the smoothness of *X*. The third term aims to ensure that the experimental validated interactions can be well preserved after the matrix completion. *A* is an adjacency matrix with binary values which is defined to clearly describe the validated DTIs; i.e., if a specific drug *D*_*i*_ is confirmed to be interacted with a target *T*_*j*_, the entity *A*(*i*, *j*) is assigned 1 or otherwise 0. Thus, the adjacency matrix *A* is with size *d* × *t*. Since *A* is with 0 − 1 values, we use itself as the indicator matrix to indicate the indices of the observed DTIs. The forth term regularized by parameter *λ* constrains that drugs with similar chemical structure are more likely to be connected with similar targets and targets with similar genomic sequence similarity are forced to have interactions with similar drugs. *DS*(*i*, *j*) represents the chemical structure similarity between drugs *D*_*i*_ and *D*_*j*_, and *TS*(*i*, *j*) represents the genomic sequence similarity between targets *T*_*i*_ and *T*_*j*_.

### 2.5. Optimization of DLGRMC

To solve the optimization problem in (3), we first transform it into the following form:

(4) minX X∗+αXF2+βA∘X−AF2+λtr⁡XTLdX+tr⁡XLtXT,

where *L*_*d*_ ∈ *ℝ*^*d*×*d*^ is the drug Laplacian matrix with *L*_*d*_ = *D*_*d*_ − *DS*, *D*_*d*_ is the diagonal matrix with *D*_*d*_(*i*, *i*) = ∑_*j*_*DS*(*i*, *j*), *L*_*t*_ ∈ *ℝ*^*t*×*t*^ is the target Laplacian matrix with *L*_*t*_ = *D*_*t*_ − *TS*, and *D*_*t*_ is the diagonal matrix with *D*_*t*_(*p*, *p*) = ∑_*q*_*TS*(*p*, *q*).

Since problem (4) contains Hadamard product of two matrices, it is hard to tackle it directly. Thus, we propose an alternative iterative algorithm to solve this problem based on Augmented Lagrange Multiplier (ALM) algorithm [49–52]. We first introduce two auxiliary variables *J* and *Z* to make the objective function separable:

(5) minX,J,Z J∗+αXF2+βA∘Z−AF2+λtr⁡XTLdX+tr⁡XLtXTs.t. X=J, X=Z.

The corresponding augmented Lagrange function of (5) is

(6) LX,J,Z,Y1,Y2,μ1,μ2=J∗+αXF2+βA∘Z−AF2+λtr⁡XTLdX+tr⁡XLtXT+Y1,X−J+μ12X−JF2+Y2,X−Z+μ22X−ZF2,

where *Y*_1_ and *Y*_2_ are the Lagrange multipliers, *μ*_1_ > 0 and *μ*_2_ > 0 control the penalties for violating the linear constraints, and 〈·, ·〉 represents the standard inner product of two matrices. Then the variables can be solved alternatively.

#### 2.5.1. Solving *J* with Other Variables Fixed

The variable *J* can be solved by the following equation with other variables fixed:

(7) minJ⁡J∗+Y1,X−J+μ12X−JF2=minJ⁡J∗+μ12X−J+Y1μ1F2,

where *J* can be solved by singular value thresholding (SVT) operator ([48]).

#### 2.5.2. Solving *Z* with Other Variables Fixed

When other variables are fixed, *Z* can be solved by minimizing following function:

(8) minZ⁡ βA∘Z−AF2+Y2,X−Z+μ22X−ZF2=minZ⁡ βA∘Z−AF2+μ22X−Z+Y2μ2F2.

Setting the derivative of (8) with respect to *Z* to zero and using properties of the Hadamard and Kronecker products, it is easy to get that *Z* can be obtained as follows:

(9) $$\begin{array}{c} R\;\mathrm{vec}\left(\;Z\;\right)=\mathrm{vec}\left(\;C\;\right), \end{array}$$

where *R* = 2*β* diag (vec⁡(*A*)) + *μ*_2_*I*, and *C* = 2*β*(*A*∘*A*) + *μ*_2_*X* + *Y*_2_. This is a simple linear system.

#### 2.5.3. Solving *X* with Other Variables Fixed

We can solve *X* by dropping other unrelated variables as follows:

(10) minX⁡ αXF2+λtr⁡XTLdX+tr⁡XLtXT+μ12X−J+Y1μ1F2+μ22X−Z+Y2μ2F2,

By setting the derivative of (10) with respect to *X* to zero, we have

(11) 2αX+2λLdX+XLt+μ1X−J+μ2X−Z+Y1+Y2=0.

Equation (11) is a Sylvester equation [53]. Since 2*α* + 2*λL*_*d*_ is strictly positive definite, (11) has stable solution for *X*.

#### 2.5.4. Updating Multipliers

We update the multipliers by

(12) Y1=Y1+μ1X−JY2=Y2+μ2X−Z.

The variables *J*, *Z*, and *X* are iteratively updated until convergence. Finally, we obtain the predicted DTIs based on the completed entities in matrix *X*. In summary, the detailed steps for solving the proposed DLGRMC model can be described by Algorithm 1. After we recover *X*, the predicted DTIs can be obtained by sorting the element values of *X* in descending order.

## 3. Results

### 3.1. Evaluation Metrics

**Figure 2:**
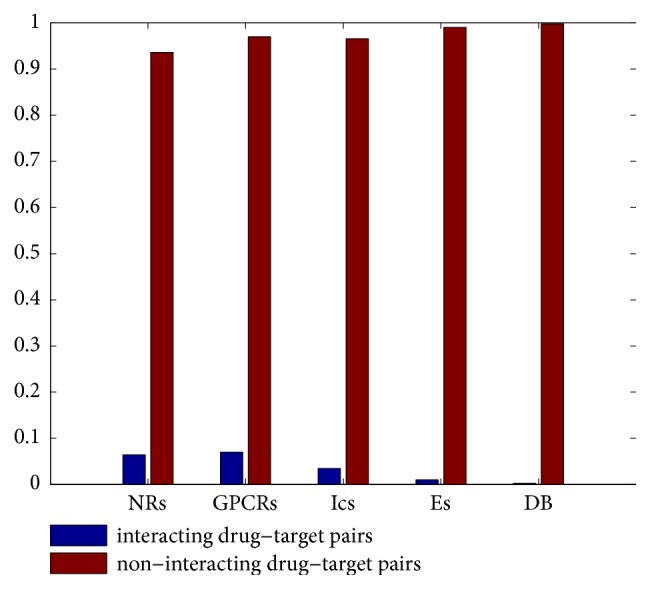
An intuitive showing of the imbalance ratio between interacting and noninteracting drug-target pairs of different datasets.

To quantitatively evaluate the performance of our method, computational experiments were conducted on the above five benchmark datasets. Similar to previous studies [27, 32, 54], the Area Under the Precision-Recall (AUPR) curve [55] and precision-recall (PR) curves were employed as the main metric for performance evaluation. AUPR can penalize the false positives more in evaluation, which is desirable here since we do not want incorrect predictions to be recommended by the prediction algorithms [55]. Before evaluating the performance of our proposed method, we give an intuitive showing of the imbalance ratio between interacting and noninteracting drug-target pairs of different datasets in Figure 2. As can be seen, the number of known drug-target interaction pairs is very small, which demonstrate the urgent need of predicting new drug-target interactions.

### 3.2. Experiments Settings

In our experiments, five existing techniques including bipartite local model using neighbor-based interaction-profile inferring (BLMNII) [32], weighted nearest neighbor profile (WNN) [54], collaborative matrix factorization (CMF) [26], graph regularized matrix factorization (GRMF) [27], neighborhood regularized logistic matrix factorization (NRLMF) [56], and label propagation with linear neighborhood information (LPLNI) [36] were used to compare with our proposed DLGRMC. We adopted 5 repetitions of 10-fold cross validation (CV) for each of the methods on different datasets. In each repetition, the observed DTIs indicator matrix *A* was divided into 10 folds. Then each fold was left out as the test set while the remaining 9 folds were treated as the training set, and the final AUPR score was the average over 5 such repetitions.

As can be seen from (3), there are three parameters that need to be turned in our proposed DLGRMC model, i.e., *α*, *β*, and *λ*. In our experiments, we have chosen them from {0.001,0.01,0.1,1, 10,100,1000} by a grid search manner, and the best results with optimal parameters were reported. As to the Gaussian kernel function for calculating the drug chemical structure similarity, we set the number of nearest neighbors *k* to be 5 and the kernel width *σ* to be 0.1. For the other methods, we set the parameters to their optimal values as recommended in the references.

Similar to previous works [9, 26, 57], we conducted CV under three different settings as follows:

- *CV*_1_: CV on drug-target pairs–random entries in *A* (i.e., drug-target pairs) were selected for testing, this setting refers to the DTIs prediction for new (unknown) drug-target pairs.
- *CV*_2_: CV on drugs–random rows in *A* (i.e., drugs) were blinded for testing, this setting refers to the DTIs prediction for new drugs.
- *CV*_3_: CV on targets–random columns in *A* (i.e., targets) were blinded for testing, this setting refers to the DTIs prediction for new targets.

Under *CV*_1_, we used 90% of elements in *A* as training data and the remaining 10% of elements as test data in each round. Under *CV*_2_, we used 90% of rows in *A* as training data and the remaining 10% of rows as test data in each round. Under *CV*_3_, we used 90% of columns in *A* as training data and the remaining 10% of columns as test data in each round.

### 3.3. DTIs Prediction Results

**Figure 3:**
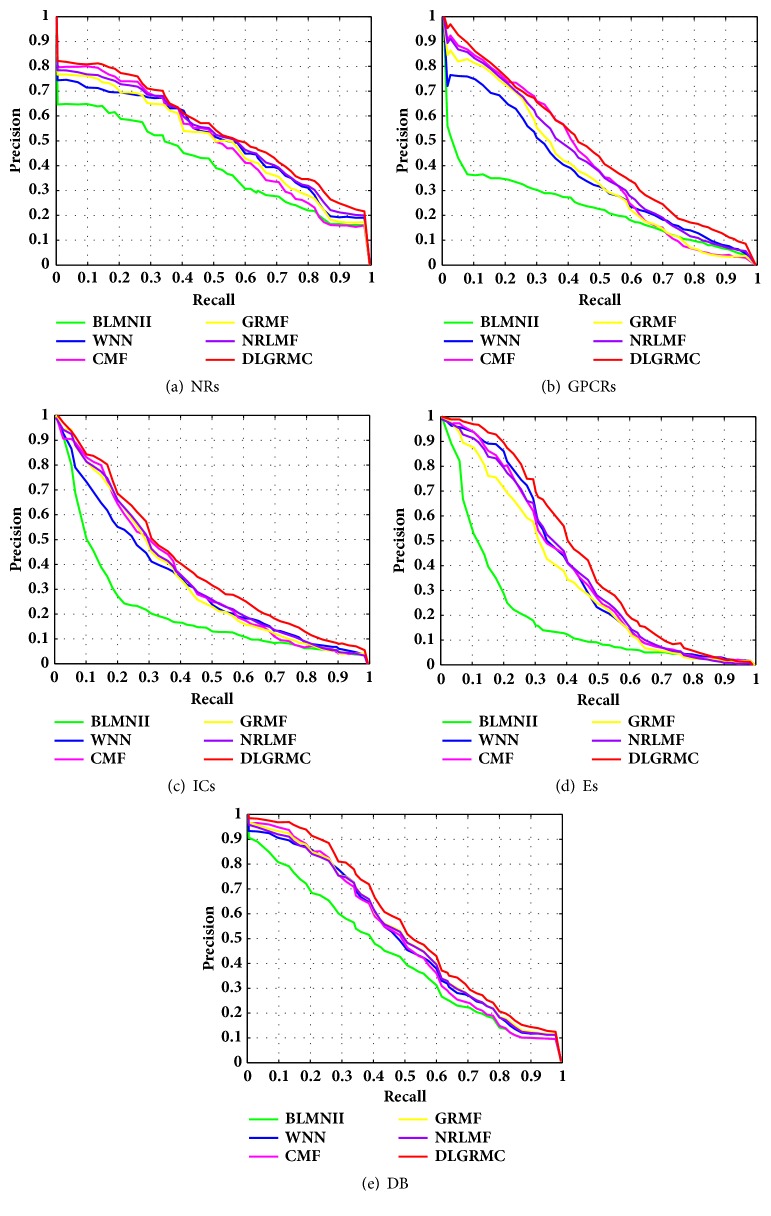
The PR curves of different methods on four datasets.

Tables 2–4 show the predicted AUPR values of different methods on different datasets under different CV settings. As can be seen, our proposed DLGRMC performs better than other methods on all of the datasets. Since the drug discovery and development aim to serve the treatment of disease, in order to predict new targets which the drugs react, we plot the precision-recall (PR) curves of the results under *CV*_3_ for all of the datasets. The plots are shown in Figure 3; the results also show the superiority of our proposed DLGRMC. We will release the related datasets, codes, and figures of our algorithm for academic research with this paper.

### 3.4. Case Study

In order to test the capacity of DLGRMC in potential DTIs prediction, we randomly chose one drug from each dataset and reported the top 10 predicted interactions of different methods under *CV*_3_. The results are shown in Tables 5–9. As can be seen, our proposed DLGRMC can successfully predict more of the experimental validated DTIs when compared with other methods, which indicates that DLGRMC is capable of predicting novel DTIs for drug development.

### 3.5. Parameter Sensitivity Analysis

**Figure 4:**
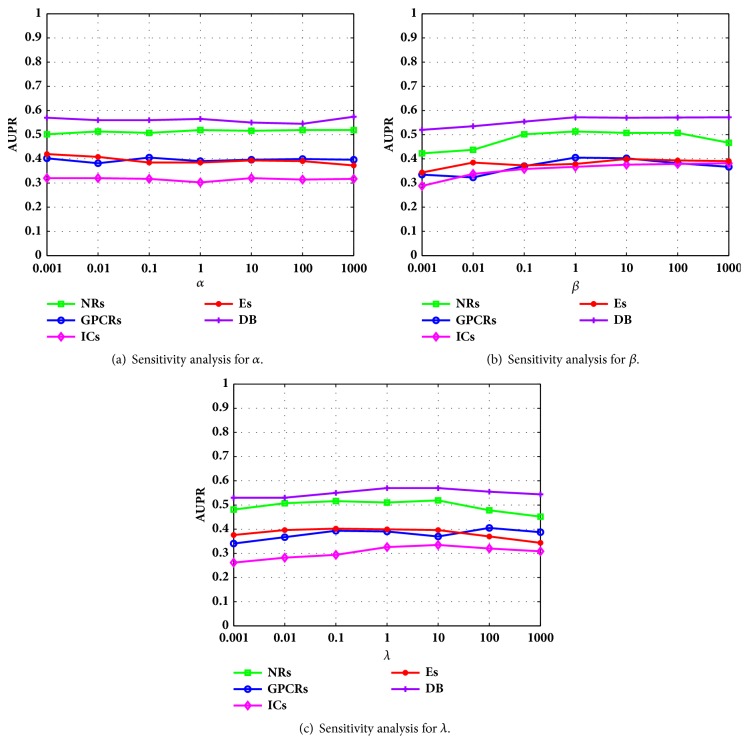
The AUPR values versus the parameter (a) *α* with *β* = *λ* = 1, (b) *β* with *α* = *λ* = 1, and (c) *λ* with *α* = *β* = 1 on different datasets.

As mentioned in Section 3.2, there are three parameters that need to be tuned for obtaining the best results. In this subsection, in order to analyse the parameter effect on the final prediction results, for each dataset, we show the AUPR values versus one of the parameters with the other two fixed.  Figure 4 plots the AUPR values of DLGRMC with different parameters on different datasets under *CV*_3_. As can be seen, DLGRMC is more sensitive to *β* and *λ* than *α*, which demonstrates the importance of the Laplacian graph regularization and the preservation of observed DTIs.

## 4. Discussion

In this paper, we propose a drug-target interaction prediction model via Laplacian graph regularized matrix completion. In detail, we transformed the task of drug-target interaction prediction into a matrix completion problem, in which the potential interactions between drugs and targets can be obtained based on the prediction scores after the matrix completion procedure. The novelties of our proposed method line in two aspects. On the one hand, during the matrix completion, the pairwise chemical structure similarities between drugs and genomic sequence similarities between drugs are fully exploited to serve the matrix completion by using a Laplacian graph regularization term. On the other hand, an indicator matrix with binary values which indicates the indices of the observed drug-target interactions is deployed to preserve the experimental confirmed interactions. We developed an alternative iterative strategy to solve the constrained matrix completion problem based on Augmented Lagrange Multiplier algorithm. The final experimental results validate the efficacy of the proposed method, and case studies demonstrate that the proposed method owns the capacity to predict potential novel drug-target interactions.

Of course, experimental results also illustrate that there is still much room for improvement since there are also missed interactions in case studies. In our recent work, only one type of representation for drugs or targets is considered. Practically, each drug or target can have multiple representations. For example, a drug can be represented by its chemical structure or by its chemical response in different cells. A protein target can be represented by its sequence or by its gene expression values in different cells. In our future work, we aim to integrate these multiview representations for drug-target interaction prediction and we believe that the prediction results can be improved with a large margin.

## Figures and Tables

**Algorithm 1:**
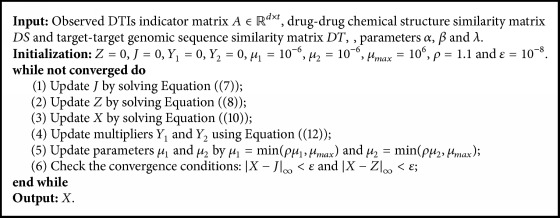
Iterative algorithm for solving DLGRMC.

**Table 1:** The statistics of drugs, targets, and interactions in each dataset.

**Datasets **	** NRs **	**GPCRs **	** ICs **	** Es **	** DB**
Drugs	54	223	210	445	1936
Targets	26	95	204	664	1609
Interactions	90	635	1476	2926	7019
Average No. of drugs per target	3.46	6.68	7.24	4.41	4.36
Average No. of targets per drug	1.67	2.85	7.03	6.58	3.63
Sparsity of the interaction matrix (%)	93.59	97.00	96.55	99.01	99.77
Percentage of drugs with only one interaction target (%)	72.22	47.53	38.57	39.78	75.20
Percentage of targets with only one interaction drug (%)	30.77	35.79	11.27	43.37	30.53

**Table 2:** Average AUPR values of different methods on different datasets under *CV*_1_ (the values following the symbol “±" are the standard deviations of 5 repetition results).

Methods	NRs	GPCRs	Ics	Es	DB
BLM-NII	0.641±0.038	0.483±0.019	0.645±0.010	0.624±0.013	0.667±0.024
WNN	0.567±0.024	0.559±0.020	0.583±0.018	0.591±0.016	0.652±0.027
CMF	0.577±0.038	0.674±0.011	0.858±0.008	0.806±0.005	0.883±0.019
GRMF	0.592±0.025	0.679±0.012	0.367±0.015	0.324±0.014	0.704±0.029
NRLMF	0.675±0.034	0.687±0.017	0.889±0.010	0.847±0.007	0.902±0.030
LGRMC	0.696±0.022	0.701±0.014	0.899±0.013	0.874±0.009	0.921±0.016

**Table 3:** Average AUPR values of different methods on different datasets under *CV*_2_ (the values following the symbol “±" are the standard deviations of 5 repetition results).

Methods	NRs	GPCRs	Ics	Es	DB
BLM-NII	0.427±0.045	0.308±0.020	0.289±0.029	0.246±0.021	0.443±0.031
WNN	0.501±0.051	0.286±0.018	0.237±0.034	0.251±0.037	0.543±0.034
CMF	0.465±0.052	0.358±0.016	0.268±0.031	0.203±0.022	0.482±0.026
GRMF	0.481±0.056	0.357±0.017	0.284±0.027	0.252±0.018	0.507±0.024
NRLMF	0.540±0.052	0.361±0.019	0.348±0.031	0.345±0.033	0.575±0.027
LGRMC	0.572±0.054	0.377±0.018	0.364±0.028	0.373±0.020	0.601±0.030

**Table 4:** Average AUPR values of different methods on different datasets under *CV*_3_ (the values following the symbol “±" are the standard deviations of 5 repetition results).

Methods	NRs	GPCRs	Ics	Es	DB
BLM-NII	0.412±0.042	0.332±0.014	0.205±0.011	0.167±0.010	0.446±0.023
WNN	0.517±0.024	0.364±0.009	0.319±0.012	0.385±0.013	0.527±0.016
CMF	0.484±0.035	0.407±0.007	0.352±0.009	0.376±0.006	0.535±0.012
GRMF	0.517±0.026	0.367±0.010	0.343±0.017	0.346±0.010	0.539±0.018
NRLMF	0.491±0.048	0.409±0.042	0.358±0.016	0.395±0.014	0.550±0.029
LGRMC	0.527±0.023	0.415±0.012	0.362±0.015	0.410±0.012	0.574±0.019

**Table 5:** The top 10 interacting targets of drug “D00094" in dataset NRs predicted by different methods (“√" denotes experimental validated targets and “×" denotes nonvalidated targets).

Rank	Targets predicted by different methods					
	BLM-NII	WNN	CMF	GRMF	NRLMF	DLGRMC
1	hsa5914 (√)	hsa190 (√)	hsa6096 (√)	hsa6257 (√)	hsa5915 (√)	hsa5914 (√)
2	hsa5915 (√)	hsa6257 (√)	hsa6257 (√)	hsa5915 (√)	hsa190 (√)	hsa5915 (√)
3	hsa6257 (√)	hsa5915 (√)	hsa5915 (√)	hsa6256 (√)	hsa6096 (√)	hsa190 (√)
4	hsa190 (√)	hsa6256 (√)	hsa190 (√)	hsa190 (√)	hsa5914 (√)	hsa6096 (√)
5	hsa6258 (√)	hsa190 (√)	hsa6256 (√)	hsa6258 (√)	hsa6097 (√)	hsa6257 (√)
6	hsa6097 (√)	hsa6097 (√)	hsa5916 (√)	hsa5916 (√)	hsa6258 (√)	hsa6256 (√)
7	hsa2099 (×)	hsa5916 (√)	hsa2104 (×)	hsa5915 (√)	hsa5916 (√)	hsa6258 (√)
8	hsa4306 (×)	hsa2908 (×)	hsa2421 (×)	hsa2101 (×)	hsa6257 (√)	hsa5916 (√)
9	hsa5465 (×)	hsa2104 (×)	hsa4306 (×)	hsa2104 (×)	hsa367 (×)	hsa2099 (×)
10	hsa2104 (×)	hsa2421 (×)	hsa9970 (×)	hsa5465 (×)	hsa4306 (×)	hsa2908 (×)

**Table 6:** The top 10 interacting targets of drug “D00255” in dataset GPCRs predicted by different methods (“√” denotes experimental validated targets and “×” denotes nonvalidated targets).

Rank	Targets predicted by different methods					
	BLM-NII	WNN	CMF	GRMF	NRLMF	DLGRMC
1	hsa147 (√)	hsa150 (√)	hsa151 (√)	hsa155 (√)	hsa155 (√)	hsa147 (√)
2	hsa148 (√)	hsa146 (√)	hsa146 (√)	hsa150 (√)	hsa147 (√)	hsa155 (√)
3	hsa146 (√)	hsa155 (√)	hsa147 (√)	hsa151 (√)	hsa146 (√)	hsa151 (√)
4	hsa150 (√)	hsa153 (√)	hsa148 (√)	hsa147 (√)	hsa150 (√)	hsa150 (√)
5	hsa1812 (×)	hsa154 (√)	hsa155 (√)	hsa154 (√)	hsa148 (√)	hsa146 (√)
6	hsa2550 (×)	hsa1234 (×)	hsa154 (√)	hsa1268 (×)	hsa2550 (×)	hsa154 (√)
7	hsa2913 (×)	hsa1241 (×)	hsa2911 (×)	hsa135 (×)	hsa3361 (×)	hsa1128 (×)
8	hsa5739 (×)	hsa3354 (×)	hsa1241 (×)	hsa2911 (×)	hsa5729 (×)	hsa2911 (×)
9	hsa7201 (×)	hsa7201 (×)	hsa3354 (×)	hsa57105 (×)	hsa9052 (×)	hsa3269 (×)
10	hsa552 (×)	hsa6751 (×)	hsa6751 (×)	hsa886 (×)	hsa2911 (×)	hsa3352 (×)

**Table 7:** The top 10 interacting targets of drug “D00110" in dataset ICs predicted by different methods (“√" denotes experimental validated targets and “×" denotes nonvalidated targets).

Rank	Targets predicted by different methods					
	BLM-NII	WNN	CMF	GRMF	NRLMF	DLGRMC
1	hsa6336 (√)	hsa11280 (√)	hsa6530 (√)	hsa6532 (√)	hsa6529 (√)	hsa6331 (√)
2	hsa6532 (√)	hsa6530 (√)	hsa6532 (√)	hsa11280 (√)	hsa6532 (√)	hsa6336 (√)
3	hsa6530 (√)	hsa6529 (√)	hsa11280 (√)	hsa6336 (√)	hsa6336 (√)	hsa6530 (√)
4	hsa11280 (√)	hsa6331 (√)	hsa6529 (√)	hsa6336 (√)	hsa6331 (√)	hsa6532 (√)
5	hsa6529 (√)	hsa6532 (√)	hsa6331 (√)	hsa6530 (√)	hsa11280 (√)	hsa11280 (√)
6	hsa2554 (×)	hsa2554 (×)	hsa6336 (√)	hsa6529 (√)	hsa9312 (×)	hsa6529 (√)
7	hsa2901 (×)	hsa9177 (×)	hsa2901 (×)	hsa1137 (×)	hsa93589 (×)	hsa1141 (×)
8	hsa3748 (×)	hsa773 (×)	hsa27012 (×)	hsa9312 (×)	hsa23704 (×)	hsa1137 (×)
9	hsa1134 (×)	hsa8514 (×)	hsa8973 (×)	hsa3762 (×)	hsa2892 (×)	hsa9312 (×)
10	hsa9177 (×)	hsa9311 (×)	hsa2560 (×)	hsa1139 (×)	hsa3756 (×)	hsa93589 (×)

**Table 8:** The top 10 interacting targets of drug “D00002" in dataset Es predicted by different methods (“√" denotes experimental validated targets and “×" denotes nonvalidated targets).

Rank	Targets predicted by different methods					
	BLM-NII	WNN	CMF	GRMF	NRLMF	DLGRMC
1	hsa216 (√)	hsa108 (√)	hsa1725 (×)	hsa196883 (√)	hsa191 (√)	hsa191 (√)
2	hsa108 (√)	hsa1725 (×)	hsa108 (√)	hsa191 (√)	hsa196883 (√)	hsa1725 (×)
3	hsa1725 (×)	hsa191 (√)	hsa2936 (√)	hsa7498 (√)	hsa108 (√)	hsa196883 (√)
4	hsa2746 (√)	hsa3939 (√)	hsa2639 (√)	hsa3033 (√)	hsa3292 (√)	hsa108 (√)
5	hsa196883 (√)	hsa3292 (√)	hsa115 (√)	hsa108 (√)	hsa3615 (√)	hsa2936 (√)
6	hsa7015 (×)	hsa349565 (√)	hsa2597 (√)	hsa7299 (×)	hsa3939 (√)	hsa3033 (√)
7	hsa4594 (×)	hsa34 (×)	hsa3156 (×)	hsa84152 (×)	hsa3818 (×)	hsa349565 (√)
8	hsa3035 (×)	hsa8435 (×)	hsa51095 (×)	hsa590 (×)	hsa5536 (×)	hsa339221 (×)
9	hsa306 (×)	hsa51095 (×)	hsa90 (×)	hsa3156 (×)	hsa34 (×)	hsa3156 (×)
10	hsa8435 (×)	hsa306 (×)	hsa761 (×)	hsa34 (×)	hsa90 (×)	hsa3991 (×)

**Table 9:** The top 10 interacting targets of drug “DB00171" in dataset DB predicted by different methods (“√" denotes experimental validated targets and “×" denotes nonvalidated targets).

Rank	Targets predicted by different methods					
	BLM-NII	WNN	CMF	GRMF	NRLMF	DLGRMC
1	P10398 (√)	P00519 (√)	P36896 (√)	P35626 (√)	O95477 (√)	Q09428 (√)
2	P36896 (√)	P35626 (√)	O43681 (√)	Q08828 (√)	P00519 (√)	P49902 (√)
3	P42684 (√)	Q9UM73 (√)	Q07912 (√)	Q9UM73 (√)	P35626 (√)	O95477 (√)
4	Q9UM73 (√)	O43681 (√)	P35626 (√)	P10398 (√)	P10398 (√)	P00519 (√)
5	O43681 (√)	P36896 (√)	P49902 (√)	P36896 (√)	P42684 (√)	P35626 (√)
6	Q07912 (√)	Q16671 (√)	Q08828 (√)	O43681 (√)	Q9UM73 (√)	Q08828 (√)
7	O95477 (√)	O95342 (√)	Q9UM73 (√)	Q07912 (√)	P36896 (√)	O14727 (√)
8	P31749 (×)	Q13131 (×)	P31749 (×)	Q15822 (×)	O43681 (√)	Q9UM73 (√)
9	P20839 (×)	P20839 (×)	P20839 (×)	P53985 (×)	Q13131 (×)	P10398 (√)
10	Q8NFJ5 (×)	P31749 (×)	P16219 (×)	P31749 (×)	O15270 (×)	P31749 (×)

## Data Availability

The datasets used in this work are publicly available at http://web.kuicr.kyoto-u.ac.jp/supp/yoshi/drugtarget/ and https://www.drugbank.ca/.
